# Association of a Healthy Lifestyle with Mortality in Older People

**DOI:** 10.21203/rs.3.rs-2541145/v1

**Published:** 2023-03-13

**Authors:** Catherine Robb, Prudence Carr, Jocasta Ball, Alice Owen, Lawrence J. Beilin, Anne B. Newman, Mark R. Nelson, Christopher M Reid, Suzanne G. Orchard, Johannes T Neumann, Andrew M. Tonkin, Rory Wolfe, John J. McNeil

**Affiliations:** Monash University; Monash University; Monash University; Monash University; University of Western Australia; University of Pittsburgh; University of Tasmania; Curtin University; Monash University; University Heart & Vascular Centre Hamburg; Monash University; Monash University; Monash University

**Keywords:** Lifestyle, Mortality, Older people, Cohort study

## Abstract

**Background:**

Unhealthy lifestyle behaviours such as smoking, high alcohol consumption, poor diet or low physical activity are associated with morbidity and premature mortality. Public health guidelines provide recommendations for adherence to these four factors, however, their impact on the health of older people is less certain.

**Methods:**

The study involved 11,340 Australian participants (median age 7.39 [Interquartile Range (IQR) 71.7, 77.3]) from the ASPirin in Reducing Events in the Elderly study, followed for a median of 6.8 years (IQR: 5.7, 7.9). We investigated whether a point-based lifestyle score based on adherence to guidelines for a healthy diet, physical activity, non-smoking and moderate alcohol consumption was associated with all-cause and cause-specific mortality.

**Results:**

In multivariable adjusted models, compared to those in the unfavourable lifestyle group, individuals in the moderate lifestyle group (Hazard Ratio (HR) 0.73 [95% CI 0.61, 0.88]) and favourable lifestyle group (HR 0.68 [95% CI 0.56, 0.83]) had lower risk of all-cause mortality. A similar pattern was observed for cardiovascular related mortality and non-cancer/non-cardiovascular related mortality. There was no association of lifestyle with cancer-related mortality. Stratified analysis indicated larger effect sizes among males, those ≤ 73 years old and among those in the aspirin treatment group.

**Conclusions:**

In a large cohort of initially healthy older people, reported adherence to a healthy lifestyle is associated with reduced risk of all-cause and cause-specific mortality.

## Introduction

The incidence of chronic disease increases with age and are major contributors to mortality amongst older individuals (1). Cardiovascular disease (CVD), cancer and dementia are the most common and costly of these diseases leading to premature mortality, but are partially preventable (2).

Common unhealthy lifestyle behaviours, such as smoking (3), high alcohol consumption (4), adverse dietary patterns (5) or low physical activity (6), have each been associated with morbidity and premature mortality in middle to older-age (7). As a result, public health authorities in various countries have provided recommendations related to these behaviours in order to preserve good health. However, it is less certain whether reported adherence to these recommendations is associated with appreciable benefit among older people (8).

Previous studies have suggested that a combination of healthy lifestyle behaviours incurs the strongest benefit in promoting healthy longevity (9) and reduced risk of premature mortality (10). A meta-analysis of 15 studies including 531,804 participants from the United States, Europe, China and Japan (mean follow-up 13.24 years) reported that up to 66% of premature deaths could be attributable to a combination of unhealthy lifestyle characteristics including smoking, high alcohol consumption, physical inactivity and a poor diet (10),(2,11,12).

Currently, the studies underpinning health guidelines and lifestyle recommendations for the wider population are mainly derived from middle-aged cohorts. The few studies focussing exclusively on older people have limited sample-sizes and/or focussed exclusively on all-cause mortality while neglecting to investigate the impacts on cause specific mortality (11, 13, 14). Clarifying the relationship between adherence and advice concerning lifestyle behaviours and mortality may help prioritise preventive advice and policy recommendations among progressively aging populations.

The Aspirin in Reducing Events in the Elderly (ASPREE) study, a cohort of initially healthy older people, is uniquely suited to investigate whether a combination of healthy lifestyle behaviours in community-dwelling older people is associated with a prolonged life span and reduced cause specific mortality (15).

## Methods

### Study population and trial design

This current analysis is based on data from the Australian participants in the ASPREE (n = 16,703), and ASPREE trial sub-sets: the ASPREE-eXTension (ASPREE-XT) study (15-17) and the ASPREE Longitudinal Study of Older Persons (ALSOP) sub-study (n = 14,892) (18). ASPREE was a large, randomised, double-blind, placebo-controlled trial investigating the efficacy of 100mg of aspirin on disability free survival in healthy men and women who were 70 years of age or older. Details of ASPREE and the primary results of the study have been published previously (15-17). Briefly, all participants were required to be in good health, with no prior cardiovascular disease events, dementia or major physical disability and expected to survive for at least five years at the time of enrolment. All participants provided written informed consent.

Following completion of the ASPREE clinical trial (2010 – June 2017), the ASPREE-XT observation follow-up period commenced (ongoing). Data in this analysis includes those collected up to the participants’ second annual ASPREE-XT visit (2020). ASPREE and ASPREE-XT were approved by the local ethics committees and is registered on clinicaltrials.gov on 24/12/2009 (NCT01038583).

The ALSOP sub-study is a longitudinal cohort study involving approximately 90% of the Australian participants in ASPREE and ASPREE-XT. Details of the ALSOP study methodology and baseline characteristics have been published elsewhere (18). Participation involved voluntary completion of a set of medical and social questionnaires mostly administered during the first year following ASPREE study enrolment, and again after three years of ASPREE participation.

### Lifestyle score

The lifestyle score was constructed based on four modifiable lifestyle factors (alcohol consumption, smoking status, physical activity and diet) known to be associated with chronic disease onset (11, 19-23). The score was created by allocating one-point for adherence to each of the 4 lifestyle behaviours defined on the basis of national and international recommendations (Table S1). The lifestyle score ranged from 0 to 4, with higher scores indicating higher adherence to healthy lifestyle recommendations. The lifestyle score was also subsequently categorised in 3 groups, as unfavourable (lifestyle score ≤ 1), moderate (lifestyle score = 2), and favourable (lifestyle score ≥ 3).

### Assessment of lifestyle factors

Full details of the assessment of the lifestyle factors and classification is described in detail in Table S1. Briefly, baseline smoking status was categorised as either current or no current smoking (including former smokers). A previous study using ASPREE data found moderate alcohol consumption to be associated with a reduced risk of CVD and all-cause mortality, as reported previously (4, 24). In the current analyses, participants were therefore categorised as either having moderate alcohol consumption or not, defined as those reporting between 51–100 grams of alcohol per week (approximating to an average of 0.7 to 1.4 standard Australian alcoholic beverages a day) at baseline, synonymous to previous cut-offs (24) and informed by current NHMRC guidelines (25). The World Health Organization (WHO) and Australian government guidelines for adults aged ≥ 65 years recommend at least 30 minutes of moderate activity at least five days per week (26, 27). Therefore, participants were categorised into either engaging in low weekly activity: no or light activity; or high: moderate or vigorous activity based on their responses at baseline. Dietary data was not available at baseline; therefore, year-three diet was utilised instead. Diet was assessed by a 49-item simple food frequency questionnaire, covering major food groups. Consumption was assessed over five predefined categories of responses ranging from “never/rarely” to “every day or several times a day”. A healthy diet was based on the consumption of at least four of seven commonly eaten food groups, following recommendations on dietary priorities for cardiometabolic health (28), and previously used elsewhere (22).

### Outcomes

Methodological details on the ascertainment of all-cause and categorisation of cause-specific mortality have been published (16, 17) and further detail is provided in Table S2. Briefly, all deaths and underlying cause were adjudicated by clinicians masked to treatment allocation and confirmed by review of at least two independent sources such as family report, clinical record or public death notice, and via a final cross check through linkage with the National Death Indices. The primary outcome was all-cause mortality. Secondary outcomes included cancer-related mortality, CVD-related mortality (including stroke, coronary and cardiovascular related death) and non-CVD/non-cancer mortality (the latter referred to as ‘other’ mortality).

### Statistical analyses

**Figure 1:**
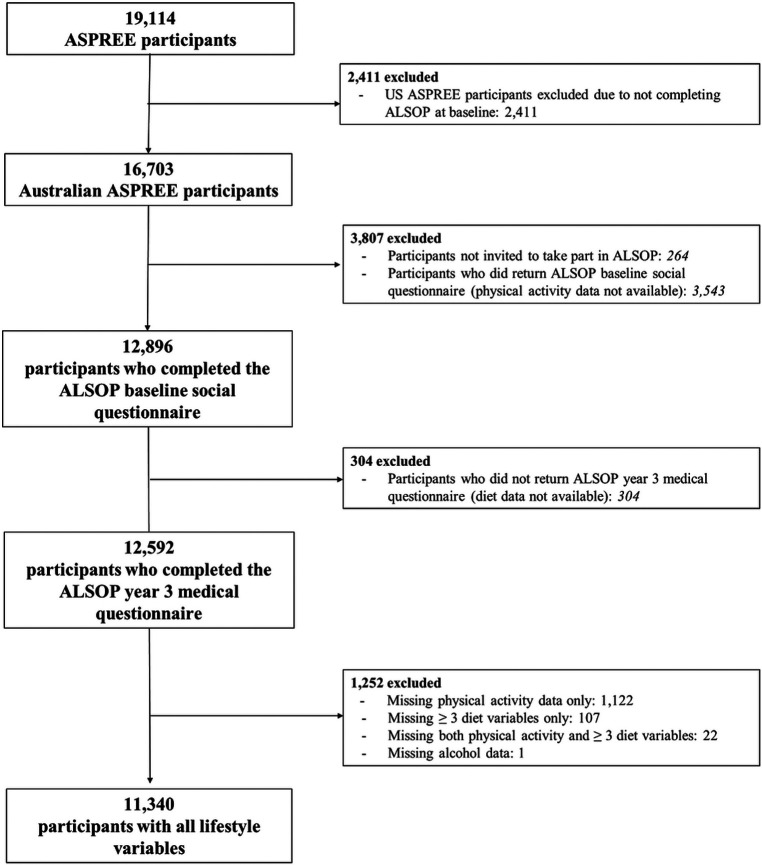
Flow of participant inclusion

Participants were included in the current analysis if they had completed both baseline and year-three ALSOP questionnaires (Fig. 1). Baseline characteristics were reported using descriptive statistics and stratified by the lifestyle score categories (unfavourable, moderate, favourable). Comparison of baseline characteristics across categories was made using the χ^2^ test, analysis of variance (ANOVA) or Kruskal- Wallis test, as appropriate.

Cox proportional hazards models were used to estimate hazard ratios (HRs) and 95% confidence intervals (CIs) for the association between the lifestyle score and all-cause and cause-specific HRs for each of cancer, CVD and ‘Other’ mortality. Competing risk Kaplan–Meier curves were plotted to illustrate the cumulative effect of lifestyle categories on all-cause and cause-specific mortality. Analyses were adjusted for age (continuous), sex (male/female), aspirin treatment allocation (100mg enteric coated aspirin/placebo) (model 1), education (≥ 12-years/< 12-years), living status (alone/with others), and socioeconomic status (IRSAD deciles, continuous) (model 2). Full details on the ascertainment of these study measurements have been described in detail previously (15, 18).

To investigate whether a priori selected factors modified the association between the lifestyle score and all-cause mortality, we performed analyses stratified by median age (≥ 74/<74 years), sex (male/female), education (≥ 12-years/< 12-years), Body Mass Index (BMI) (≥ 25 vs < 25 kg/m^2^), baseline diagnosis of type-2 diabetes (yes/no) and baseline diagnosis of hypertension (yes/no) and aspirin treatment allocation. Interaction was tested by including a cross-product term along with the main effect terms in the models. In exploratory analyses, we investigated the association between the individual lifestyle factors with all-cause and cause-specific mortality and combinations of individual lifestyle factors with all-cause mortality.

In sensitivity analyses we explored alternative definitions of adherence to the lifestyle factors to assess whether significant differences in associations with all-cause mortality would result. For example, there is debate over whether the ‘protective’ effect of moderate alcohol consumption is real or spurious due to residual confounding (29). Therefore, we created a second variable allocating participants to either moderate/low/never vs high. This variable was associated with all-cause mortality alone and as part of the lifestyle score. Second, we excluded former alcohol consumers and former smokers (who quit <15-years ago), who may have stopped due to various health reasons, possibly introducing bias from reverse causality. Third, owing to the utility of year-three diet, in sensitivity analyses, we tested the association between the same lifestyle score, but now comprised of year-three smoking, alcohol, physical activity and diet, with all-cause mortality. Assuming lifestyle does not change significantly, and owing to the fact that participants must have survived the first three years to be included in the analysis, we hypothesise that the HRs will remain equivocal to those utilising the baseline lifestyle score.

Statistical analyses were conducted using Stata (version 17; College Station, TX: StataCorp LLC). A two-sided *p*-value of ≤ 0.05 was considered statistically significant. The proportional hazards assumption was assessed and met using log-log Kaplan-Meier survival plots.

## Results

A total of 11,340 Australian participants were included in the current analysis, and followed for a median of 6.8 years (IQR: 5.7, 7.9) years. At study entry, the median age was 73.9 (IQR 71.7–77.3) years and 54.2% were female. A total of 702 (6.2%) participants died during the follow-up period.

### Baseline characteristics

Baseline characteristics of included participants according to the lifestyle score categories as well as by all-cause mortality are shown in Table 1 and Table S3, respectively. Overall, 20.4% of participants adhered to one or no lifestyle factors (unfavourable lifestyle), 44.2% of participants adhered to two lifestyle factors (moderate lifestyle) and 35.3% adhered to three or four lifestyle factors (favourable lifestyle). There was a significantly higher proportion of younger participants, females, those living with others and those with a higher education in the favourable lifestyle group.

A higher proportion of individuals with diagnosed vascular risk factors (i.e. hypertension, diabetes, dyslipidaemia), who were on antihypertensives and statins, who were pre-frail/frail, with depressive symptoms and/or higher BMI, waist circumference and systolic blood pressure were in the unfavourable lifestyle group. There were no differences across lifestyle groups based on aspirin treatment allocation. Description and prevalence of the lifestyle factors in the population are shown in Table S1. With regard to adherence to healthy lifestyle factors, 97.5% reported no current smoking, 22.3% reported moderate alcohol consumption, 33.3% met the ‘healthy diet’ criteria and 66.9% of participants reported engagement in weekly moderate/vigorous physical activity.

### Association between lifestyle and mortality

**Figure 2:**
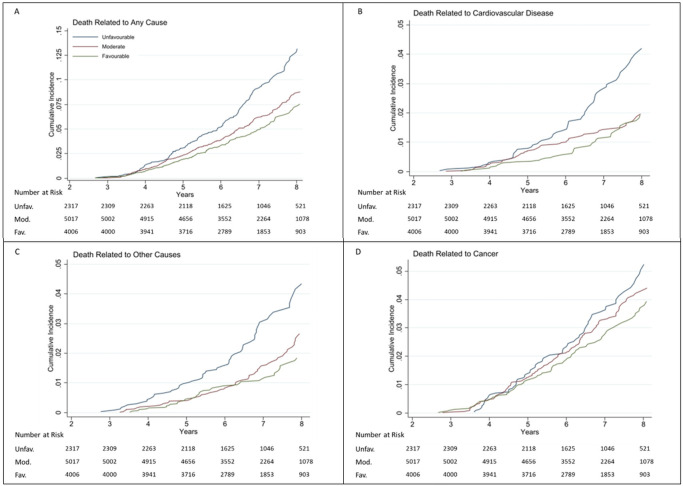
Cumulative Incidence of all-cause and cause specific mortality by lifestyle categories Shown is the cumulative incidence of death due to any cause and death according to major underlying causes (cancer, cardiovascular disease and ‘other’ non-CVD/non-cancer cause). For each cause of death, the cumulative incidence was based on a competing-risks model, stratified by lifestyle categories, with the remaining causes of death as competing events. Abbreviations: Unfav., unfavourable lifestyle category; Mod., moderate lifestyle category; Fav., favourable lifestyle category. Years = years since baseline visit.

Rates of all-cause mortality and cause-specific mortality are shown in Table 2, Table 3 and Figure. For all-cause mortality, compared to participants with no or one healthy lifestyle factors, the multivariable adjusted HRs were 0.73 (95% CI 0.61, 0.88) for two factors, 0.70 (95% CI 0.57, 0.86) for three factors, and 0.56 (95% CI 0.37, 0.86) for four factors, *p*-trend < 0.0001 (Table 2; Fig. 2A). When evaluated as a continuous variable, each additional lifestyle factor was associated with a 16% lower risk of all-cause mortality (HR for a one-point increase: 0.78 [95% CI 0.71, 0.87]). A similar association was observed for CVD-related (Table 3, Fig. 2B) and ‘Other’ mortality (Table 3, Fig. 2C).

The same dose-response association was observed when the lifestyle scores were divided into three categories as shown in Table 2, Fig. 2A (all-cause mortality) and Table 3, Fig. 2B-C (cause-specific mortality). In multivariable adjusted models, compared with those in the unfavourable lifestyle group, individuals in the moderate lifestyle group had a 27% lower risk of all-cause mortality [HR 0.73 (95% CI 0.61, 0.88]), and individuals in the favourable lifestyle group had a 32% lower risk of all-cause mortality (HR 0.59 [95% CI 0.47, 0.73]), *p*-trend < 0.001 (Table 2; Fig. 2A). In absolute terms, among 1000 individuals in the unfavourable lifestyle group, a crude total of 39 deaths could be averted during the median follow-up period of 6.8 years if they were in the favourable lifestyle group.

With regard to risk of CVD and ‘Other’ mortality, the same dose-response association was observed, with larger effect size for CVD mortality (Table 3; Fig. 2B-C). There was no association between lifestyle groups and cancer-related mortality (Table 3; Fig. 2D).

### Additional analyses

Additional analyses of specific lifestyle characteristics are presented in Tables S4-S6. Each individual lifestyle factor was associated with a lower risk of all-cause mortality, CVD mortality and ‘Other’ mortality. Specifically, effect sizes of each lifestyle factor on risk of all-cause mortality were highest and statistically significant for smoking status (HR 0.42 [95% CI 0.30, 0.59]) and physical activity (HR 0.84 [95% CI 0.72, 0.98]), with non-significant trends for alcohol consumption (HR 0.92 [95% CI 0.62, 1.37]) and diet (HR 0.90 [95% CI 0.76, 1.06]). Similar trends were observed for CVD mortality and ‘Other’ mortality.

Further analyses according to different combinations of two, three and four healthy lifestyle factors prevalent in at least 2% of the population compared to no or one healthy lifestyle factors are shown in Table S7. None of the observed associations were as protective of all-cause mortality as the combination of 4 factors (HR 0.57 [95% CI 0.37, 0.86]).

Table S8 shows results of the stratified analyses by selected health, demographic and anthropometric factors on the association between the lifestyle score categories and all-cause mortality. No statistically significant differences were observed in the associations between the healthy lifestyle score categories and all-cause mortality by age, sex, education, BMI, diabetes, hypertension and aspirin treatment. Nonetheless, trends indicate larger effect sizes among males, those at or below the median age of 73-years-old and among those in the aspirin treatment group.

### Sensitivity analyses

Results from the various sensitivity analyses are presented in the Tables S9-S12, and page 14 of the supplement. Briefly, excluding former smokers/drinkers (Table S9), the alternative categorisation of alcohol consumption (supplement p 14) and the alternative lifestyle score (Table S10 and S11) did not alter the results. Multivariable adjusted HRs on the association between the year-three lifestyle score categories and risk of all-cause mortality remained equivocal (Table S12).

## Discussion

In this cohort of 11,340 community dwelling healthy older Australians, we examined the association between a healthy lifestyle score and all-cause mortality, cancer-related mortality, CVD-related mortality and ‘other’ causes over a median follow-up time of 6.8 years (IQR: 5.7, 7.9). We found that a healthy lifestyle score at baseline, comprising of four common and potentially modifiable lifestyle factors (non-smoking, moderate alcohol consumption, a healthy dietary pattern and physical activity) was associated with prolonged lifespan in a dose-response relationship, such that each additional lifestyle factor was associated with a 16% lower risk of all-cause mortality, 25% lower risk of CVD mortality and 22% lower risk of ‘Other’ mortality. The current data found no association between lifestyle groups and cancer-related mortality. The benefits of a healthy lifestyle on all-cause mortality had larger effect sizes among males, those ≤ 73 years old and among those in the aspirin treatment group, although interactions were not statistically significant.

This is one of the largest and most comprehensive studies, conducted exclusively within community dwelling older people, reporting the association between a lifestyle composite score based on adherence to international health behaviour recommendations and all-cause plus cause-specific mortality. The results are largely in agreement with previous reports investigating different combinations of healthy lifestyle characteristics. A similar study of older Chinese people (n = 11,224, aged 65–90 years) reported that, compared to those without any unhealthy factors, those who had a high BMI, poor sleep, unhealthy diet, no physical activity, consumed alcohol and currently smoked were 1.34 (95% CI 1.02, 1.76) times more likely to die from any cause over a ten-year follow-up period (14). Another 18-year follow-up study of Swedish older adults (75 +years of age; n = 1,810) reported a median survival of 5.4 years longer among those who had a healthy BMI, never smoked or drank alcohol, engaged in leisure activities and moderate levels of physical activity versus those who did not (13). “The healthy aging: a longitudinal study in Europe” (HALE) study (n = 1,507) reported that 70 to 90-year-old community-dwelling people who did not smoke, consumed a Mediterranean diet, reported moderate alcohol consumption and was physically active had a 50% lower rate of all-cause and cause-specific mortality over 10-years, including CVD and cancer-related mortality (11). Similar protective effects of a composite lifestyle score, typically including at least diet, physical activity, smoking and alcohol, have been reported among middle-aged cohorts from different counties including Japan (30), China (12), United States of America (USA) (2), Australia (31) and the United Kingdom (UK) (32).

Results reported here and previously, provide compelling evidence to suggest that individuals reporting a healthy lifestyle in older age have a significantly reduced risk of earlier mortality. The results also demonstrate that current international recommendations for moderate physical activity, no smoking, a healthy dietary pattern and moderate alcohol consumption may still provide a useful predictor of longevity among this older aged cohort.

We found no relationship between a healthy lifestyle on risk of overall cancer-related death, which is contrary to findings previously reported in the HALE study as well as among studies of younger cohorts (11). Although, it is possible that the lack of a broader link with cancer reflects the very small percentage of current smokers in ASPREE. The HALE study was conducted via survey only and among a demographic born up to 40-years earlier than ASPREE participants.

Some methodological points may impact the conclusions of this study. In order to construct the healthy lifestyle score, we dichotomised each lifestyle factor according to pre-defined cut-off points. Different threshold values may have resulted in different risk estimates. However, the choice of cut-off was largely based on national and international public health recommendations (25-28). In sensitivity analyses we trialled different cut-offs and multi-levels but the results remained largely unchanged (Tables S10, S11 and supplement p 14). The approach of designating compliance versus non-compliance allows a simple objective classification to assess the health impact of lifestyle and can inform a clear public health message. Future modifications of this approach may involve differential weighting of the health impact of each lifestyle measure.

Owing to the absence of baseline dietary data, we utilised year-three dietary data as an alternative replacement within the baseline lifestyle score. It is not certain whether dietary behavior had significantly changed over this three-year period. Dietary changes can occur in older people due to factors such as oral health, income, marital status, medication or change of residence (33). Nonetheless, as healthy lifestyle habits are characteristic of a person’s way of living, and given ASPREE is an especially healthy cohort, majority of participants were unlikely to show a substantial change in general dietary habits over a three-year period (34). Furthermore, the year-three lifestyle score was associated with all-cause and cause-specific mortality with similar effect size to associations between baseline lifestyle and mortality, sanctioning this assumption.

Finally, given we do not have detailed information about mid-life lifestyle behaviour in the ASPREE cohort, we cannot confirm whether observed associations are not driven by behaviour earlier in life. Healthy lifestyle behaviours in older age may reflect a long-standing approach to healthy living which, in turn, may be driving these observations. Our results still, however, highlight the benefits of identifying healthy lifestyle factors as predictors of likely future mortality, even among already healthy older people.

### Strengths and limitations

There are several strengths of our study. ASPREE is a well characterised, large and contemporary cohort of older people who had reached age 70-years or more in relatively good health (18). Furthermore, rigorous methods for the ascertainment of cause-specific mortality ensured highly accurate endpoints. The investigation of not only all-cause but cause-specific mortality is a further strength.

There are also several potential limitations. First, the ASPREE cohort is comprised of initially healthy volunteers for a clinical trial who are more likely to be attentive to maintaining a healthy lifestyle, hence, may represent a healthier sample of older people compared with the general population. Second, the cohort is largely Caucasian, educated and drawn from communities with access to universal healthcare as reflected by the extensive use of preventive medications including statins (in 30%) and antihypertensive agents (in 51%). Therefore, our results may not be applicable among other socioeconomic and ethnic groups as well as among those residing in lower-to middle-income countries. Third, due to the progressive nature of noncommunicable disease leading to death, with declining function often preceding and possibly influencing lifestyle behaviour, we cannot rule out reverse causality as a partial explanation for these observations. Nonetheless, although survivor bias is a common limitation in healthy cohort studies, our censoring of death events at three-years may also help to mitigate reverse causality.

Finally, although potential confounders were considered in multivariable analyses, residual confounding cannot be ruled out. Furthermore, other unmeasured lifestyle and environmental factors may also play a role in determining risk of death. However, demonstrating that these four common lifestyle behaviors are associated with prolongation of an individual’s lifespan remains an important public health message.

## Conclusion

In a well-characterised population of healthy older people, moderate exercise, a healthy dietary pattern, moderate alcohol consumption and non-smoking was associated with 44% reduced risk of all-cause mortality, when compared to those complying to ≤ 1 healthy lifestyle factor. Previous multi-lifestyle interventions among healthy older people has proven beneficial in reducing risk for CVD and cognitive decline (35-37), whereas evidence from single domain lifestyle interventions are less convincing (38). Furthermore, these studies, plus others (39), indicate that simple and effective methods for lifestyle modification is possible among older people. Findings here further suggest the importance of engaging or continuing to engage in multiple healthy lifestyle behaviours in older age and may encourage further multi-lifestyle interventions at both the population level as well as on an individual level.

## Figures and Tables

**Table 1:** Baseline characteristics of the study population according to lifestyle score categories

Characteristics	Overall cohort (n = 11,340)	Lifestyle Score Categories			p- value
		Unfavourable (n = 2,317)	Moderate (n = 5,017)	Favourable (n = 4,006)	
Age (years), median (IQR)	73.9 (71.7–77.3)	74.5 (71.9–78.4)	74.0 (71.7–77.4)	73.4 (71.5–76.6)	< 0.001
Male sex, n (%)	5,190 (45.8%)	938 (40.5%)	2,428 (48.4%)	1,824 (45.5%)	< 0.001
Living alone, n (%)	3,468 (30.6%)	786 (33.9%)	1,530 (30.5%)	1,152 (28.8%)	< 0.001
<12 years of education, n (%)	5,380 (47.4%)	1,225 (52.9%)	2,510 (50.0%)	1,645 (41.1%)	< 0.001
IRSAD decile, median (IQR)	6 (4–9)	6 (3–9)	6 (4–9)	7 (4–9)	< 0.001
Aspirin allocation, n (%)	5,647 (49.8%)	1,131 (48.8%)	2,519 (50.2%)	1,997 (49.9%)	0.537
Medical history, n (%)					
Diabetes	1,040 (9.2%)	292 (12.6%)	459 (9.2%)	289 (7.2%)	< 0.001
Hypertension	8,392 (74.0%)	1,867 (80.6%)	3,723 (74.2%)	2,802 (70.0%)	< 0.001
Dyslipidaemia	7,648 (67.4%)	1,579 (68.2%)	3,418 (68.1%)	2,651 (66.2%)	0.104
Pre-frailty	3,870 (34.1%)	1,010 (43.6%)	1,715 (34.2%)	1,145 (28.6%)	< 0.001
Frailty	144 (1.3%)	64 (2.8%)	57 (1.1%)	23 (0.6%)	< 0.001
Depressive symptoms	972 (8.6%)	271 (11.7%)	404 (8.1%)	297 (7.4%)	< 0.001
Prescribed medications, n (%)					
Statins	3,408 (30.1%)	804 (34.7%)	1,537 (30.6%)	1,067 (26.6%)	< 0.001
Antihypertensives	5,821 (51.3%)	1,397 (60.3%)	2,566 (51.2%)	1,858 (46.4%)	< 0.001
Physical Examination					
BMI kg/m^2^, mean (SD)	27.9 (4.5)	29.2 (5.1)	27.9 (4.3)	27.2 (4.1)	< 0.001
Waist Circumference (cm), mean (SD)	96.8 (12.4)	100.0 (13.2)	96.9 (12.1)	94.9 (11.9)	< 0.001
Systolic BP (mm Hg), mean (SD)	139.6 (16.2)	140.6 (16.3)	139.6 (16.2)	138.9 (16.2)	< 0.001
Diastolic BP (mm Hg), mean (SD)	77.3 (9.8)	77.6 (10.1)	77.2 (9.9)	77.1 (9.6)	0.221
Pathology					
HDL (mmol/L), mean (SD)	1.6 (0.5)	1.5 (0.4)	1.6 (0.5)	1.6 (0.5)	0.002
Non-HDL (mmol/L), mean (SD)	3.7 (0.9)	3.7 (1.0)	3.7 (0.9)	3.7 (0.9)	0.121
Creatinine (μmol/L), mean (SD)	79.7 (19.0)	80.4 (20.8)	80.4 (18.5)	78.4 (18.3)	< 0.001
eGFR (mL/min/1.73m^2^), median (IQR)	74.3 (64.0-84.2)	73.2 (61.3–83.5)	73.8 (63.6–84.0)	75.4 (65.6–84.9)	< 0.001
Healthy lifestyle factors, n (%)					
No current smoking	11,050 (97.4%)	2,106 (90.9%)	4,945 (98.6%)	3,999 (99.9%)	< 0.001
Regular physical activity	7,591 (66.9%)	106 (4.6%)	3,719 (74.1%)	3,766 (94.0%)	< 0.001
Healthy diet	3,779 (33.3%)	16 (0.7%)	916 (18.3%)	2,847 (71.1%)	< 0.001
Moderate alcohol consumption	2,525 (22.3%)	17 (0.7%)	454 (9.1%)	2,054 (51.3%)	< 0.001

Abbreviations: n, sample size; IQR, interquartile range; SD, standard deviation; cm, centimetres; mmol/L, millimoles per litre; μmol/L, micromoles per litre; mL/min, milliliter per minute; eGRF, estimated glomerular filtration rate; IRSAD, Index of Relative Socio-economic Advantage and Disadvantage (area-level socioeconomic status; higher score = less disadvantage); BMI, Body Mass Index; BP, blood pressure.

a column totals may not add up to 100% due to missing observations: IRSAD, 24 (0.21%); BMI, 52 (0.46%); Waist circumference, 101 (0.89%); HDL, 288 (2.54%); Non-HDL, 288 (2.54%); Creatinine, 332 (2.93%); eGFR, 332 (2.54%).

**Table 2:** Hazard ratios of all-cause mortality according to healthy lifestyle score

				Hazard Ratio (95% CI)^1^	
	N 11,340	No. Event	Incident rate per 1000 py	Model 1	Model 2
**Healthy lifestyle score**					
0 or 1	2,317	205	13.22	1 [Reference]	1 [Reference]
2	5,017	299	8.89	0.73 (0.61, 0.87)	0.73 (0.61, 0.88)
3	3,358	173	7.69	0.68 (0.56, 0.84)	0.70 (0.57, 0.86)
4	648	25	5.69	0.53 (0.35, 0.81)	0.56 (0.37, 0.86)
Per 1-point increase				0.82 (0.75, 0.90)	0.84 (0.76, 0.92)
**Healthy lifestyle score categories**					
Unfavourable (≤ 1)	2,317	205	13.22	1 [Reference]	1 [Reference]
Moderate (2)	5,017	299	8.89	0.73 (0.61, 0.87)	0.73 (0.61, 0.88)
Favourable (≥ 3)	4,006	198	7.36	0.66 (0.54, 0.80)	0.68 (0.56, 0.83)
p-value for trend				0.001	0.001

1 Model 1 adjusted for age, sex and aspirin treatment allocation; Model 2 adjusted for model 1 plus living status, education, socioeconomic status

**Table 3:** Hazard ratios of cause specific mortality according to healthy lifestyle score

				Hazard Ratio (95% CI)^1^	
Healthy lifestyle score categories	N 11,340	No. Event	Incident rate per 1000 py	Model 1	Model 2
**Cancer mortality** ^2^					
Unfavourable (≤ 1)	2,317	80	5.16	1 [Reference]	1 [Reference]
Moderate (2)	5,017	151	4.49	0.91 (0.69, 1.19)	0.92 (0.70, 1.20)
Favourable (≥ 3)	4,006	106	3.94	0.86 (0.64, 1.15)	0.87 (0.65, 1.17)
p-value for trend				0.30	0.37
**Cardiovascular mortality** ^3^					
Unfavourable (≤ 1)	2,317	62	4.00	1 [Reference]	1 [Reference]
Moderate (2)	5,017	69	2.05	0.58 (0.41, 0.81)	0.58 (0.42, 0.83)
Favourable (≥ 3)	4,006	44	1.64	0.51 (0.35, 0.75)	0.55 (0.37, 0.81)
p-value for trend				0.001	0.002
**‘Other’ mortality** ^4^					
Unfavourable (≤ 1)	2,317	63	4.06	1 [Reference]	1 [Reference]
Moderate (2)	5,017	78	2.32	0.63 (0.45, 0.88)	0.63 (0.45, 0.89)
Favourable (≥ 3)	4,006	48	1.79	0.54 (0.37, 0.79)	0.55 (0.38, 0.81)
p-value for trend				0.002	0.002

1 Model 1 adjusted for age, sex and aspirin treatment allocation; Model 2 adjusted for model 1 plus living status, education, socioeconomic status

2 Per one-point increase in lifestyle score, the full-adjusted HR for Cancer mortality was 0.91 (95% CI 0.80, 1.04), p = 0.17

3 Per one-point increase in lifestyle score, the full-adjusted HR for CVD mortality is 0.75 (95% CI 0.62, 0.91), p = 0.004

4 Per one-point increase in lifestyle score, the full-adjusted HR for ‘Other’ mortality was 0.78 (95% CI 0.65, 0.94), p = 0.01

## Data Availability

Upon official request and submission of an expression of interest to the ASPREE data management team, with support from the PI (Professor John McNeil), data may be made available. Deidentified participant data would be accessed via a secure remote server with an accompanying data dictionary and manual including all source data worksheets. Upon submission of the expression of interest, data can be made available within a timely manner.

## Abbreviations

HR: Hazard Ratio
IQR: Interquartile range
SD: Standard Deviation
CI: Confidence Interval
CVD: Cardiovascular Disease
ASPREE: Aspirin in Reducing Events in the Elderly study
ASPREE-XT: ASPREE-eXTension study
ALSOP: ASPREE Longitudinal Study of Older Persons sub-study
WHO: World Health Organisation
ANOVA: Analysis of Variance
IRSAD: Index of Relative Socio-economic Advantage and Disadvantage
BMI: Body Mass Index
HALE: Healthy Aging: a Longitudinal study in Europe
USA: United States of America
UK: United Kingdom
eGFR: estimated Glomerular Filtration Rate
BP: Blood pressure
